# Long-read assays shed new light on the transcriptome complexity of a viral pathogen

**DOI:** 10.1038/s41598-020-70794-5

**Published:** 2020-08-14

**Authors:** Dóra Tombácz, István Prazsák, Zsolt Csabai, Norbert Moldován, Béla Dénes, Michael Snyder, Zsolt Boldogkői

**Affiliations:** 1 Department of Medical Biology, Faculty of Medicine, University of Szeged, Szeged, 6720 Hungary; 2 Veterinary Diagnostic Directorate of the National Food Chain Safety Office, Budapest, 1143 Hungary; 3 Department of Genetics, School of Medicine, Stanford University, Stanford, CA 94305 USA

**Keywords:** Pox virus, RNA sequencing, Transcriptomics

## Abstract

Characterization of global transcriptomes using conventional short-read sequencing is challenging due to the insensitivity of these platforms to transcripts isoforms, multigenic RNA molecules, and transcriptional overlaps. Long-read sequencing (LRS) can overcome these limitations by reading full-length transcripts. Employment of these technologies has led to the redefinition of transcriptional complexities in reported organisms. In this study, we applied LRS platforms from Pacific Biosciences and Oxford Nanopore Technologies to profile the vaccinia virus (VACV) transcriptome. We performed cDNA and direct RNA sequencing analyses and revealed an extremely complex transcriptional landscape of this virus. In particular, VACV genes produce large numbers of transcript isoforms that vary in their start and termination sites. A significant fraction of VACV transcripts start or end within coding regions of neighbouring genes. This study provides new insights into the transcriptomic profile of this viral pathogen.

## Introduction

Members of the *Poxviridae* family infect various vertebrate and invertebrate host species^1^. Most notably, the variola virus is the causative agent of smallpox^2^. VACV has roughly 90% sequence homology with variola. The VACV virion contains 195 kilobase pairs (kbp) of double-stranded DNA comprising at least 200 open reading frames (ORFs), twelve of which are present in terminal repeats^3–5^. This viral genome encodes enzymes for DNA and RNA synthesis, transcription factors, and for enzymes that cap^6^ and polyadenylate^7^ RNA molecules. All of these proteins allow VACV replication in the cytoplasm of host cell.

Viral gene expression is regulated by stage-specific transcription factors that specifically bind to promoters of early (E), intermediate (I), and late (L) genes^8–11^. The complete transcription machinery is already packaged in the VACV virion allowing E genes to be expressed immediately after entering the cell, when the viral genome is still encapsidated^4^. Subsequently, DNA replication occurs which is followed by the expression of I and then L genes classes. Synthesis of I mRNAs is dependent on de novo expression of E viral proteins, whereas synthesis of mRNAs from L genes requires the expression of certain E and I genes. During the last stage of infection, newly assembled virus particles egress from host cells. E genes encode proteins that synthesize DNA and RNA molecules, and others that play roles in virus-host interactions; whereas post-replicative (PR) genes (I and L genes) mainly specify structural elements of the virus^12^. VACV genes belonging in the same kinetic class have been shown to exhibit a strong preference for co-localization^13^. Namely, E genes are reportedly clustered near genomic termini and are transcribed in the same direction^14^, whereas I and L genes are clustered at the central part of the viral DNA^15^. Using genome tiling arrays, Assarsson and colleagues demonstrated that 35 viral genes are expressed in immediate-early (IE) kinetics^13^. However, this terminology has not become widely accepted. In other DNA viruses, such as herpesviruses^16^ and baculoviruses^17^, IE RNAs are expressed in the absence of de novo protein synthesis, whereas E and L RNAs are dependent on the synthesis of IE proteins. According to this classification, all VACV E transcripts should belong to the IE kinetic class^18^. An alternative classification by Yang et al. categorized early VACV gene transcripts into two classes, E1.1 and E1.2.^19^, and defined the ensuing expression kinetics according to higher affinity of transcription factors for E1.1 than for E1.2 promoters. The three classes of genes have distinctive promoters^10^. The consensus transcription termination sequence of E transcripts (UUUUUNU) is non-functional in PR transcripts, which mostly have polymorphic 3′ ends^20,21^.

Many PR and a few E transcripts have 5′ poly(A) tails (PAT). PATs are supposedly generated by RNA polymerase slippage onto adjacent thymines on the DNA strand^22^. In addition to genome tiling analyses^23^, RNA-Seq^19^, ribosome profiling^12,24^, and microarrays^25,26^ have also been used for VACV transcriptome profiling. Yang and colleagues mapped 118 E genes and 93 PR genes using a short-read sequencing (SRS) technique^16^, and in a later study, they distinguished 53 I and 38 L genes among the PR genes^17^. Ribosome profiling analysis also confirmed canonical translational initiation sites (TISs) and demonstrated that additional TISs occur mostly within the ORFs. These TISs were also detected in 5′-untranslated (UTRs) and intergenic regions, as well as on complementary DNA strands^12^. However, because most of these ORFs were short, the authors doubted their biological relevance. In another study by Yang and colleagues, transcript abundance was highly correlated with ribosome-protected reads, suggesting that translation is largely regulated by mRNA abundance^12^. The techniques used in the above mentioned studies cannot detect full-length RNA molecules, and hence fail to provide a comprehensive assessment of the viral transcriptome. Although deep SRS provides satisfactory sequencing depth and coverage for global transcriptome profiling, the resulting RNA assemblies are incomplete, leading to inadequate annotations.

The major limitations in VACV transcriptome profiling relate to multiple transcriptional read-throughs that generate a complex meshwork of overlapping RNAs and to the large variation in transcriptional end sites (TESs). Additionally, the extensive use of alternative promoters leads to a large diversity of transcription start sites (TSSs). Hence, conventional techniques that fail to read entire RNA molecules have considerably underestimated the complexity of the poxvirus transcriptome. Earlier, we used two long-read sequencing (LRS) platform Pacific Biosciences (PacBio) and Oxford Nanopore Technologies (ONT)] to profile the transcriptomes of herpesviruses, including pseudorabies virus^27,28^, herpes simplex virus type 1^29,30^, varicella-zoster virus^31^, and human cytomegalovirus^32,33^. These analyses identified polycistronic RNAs, transcript isoforms, and transcriptional overlaps, and facilitated the kinetic characterization of viral transcripts^34,35^.

Herein, we report the transcriptomic analyses of the Western Reserve (WR) strain of VACV using PacBio RSII and Sequel platforms, and ONT MinION platform for cDNA, direct RNA (dRNA), and Cap-sequencing (Cap-Seq).

## Results

### Long-read transcriptome sequencing of vaccinia virus

**Figure 1:**
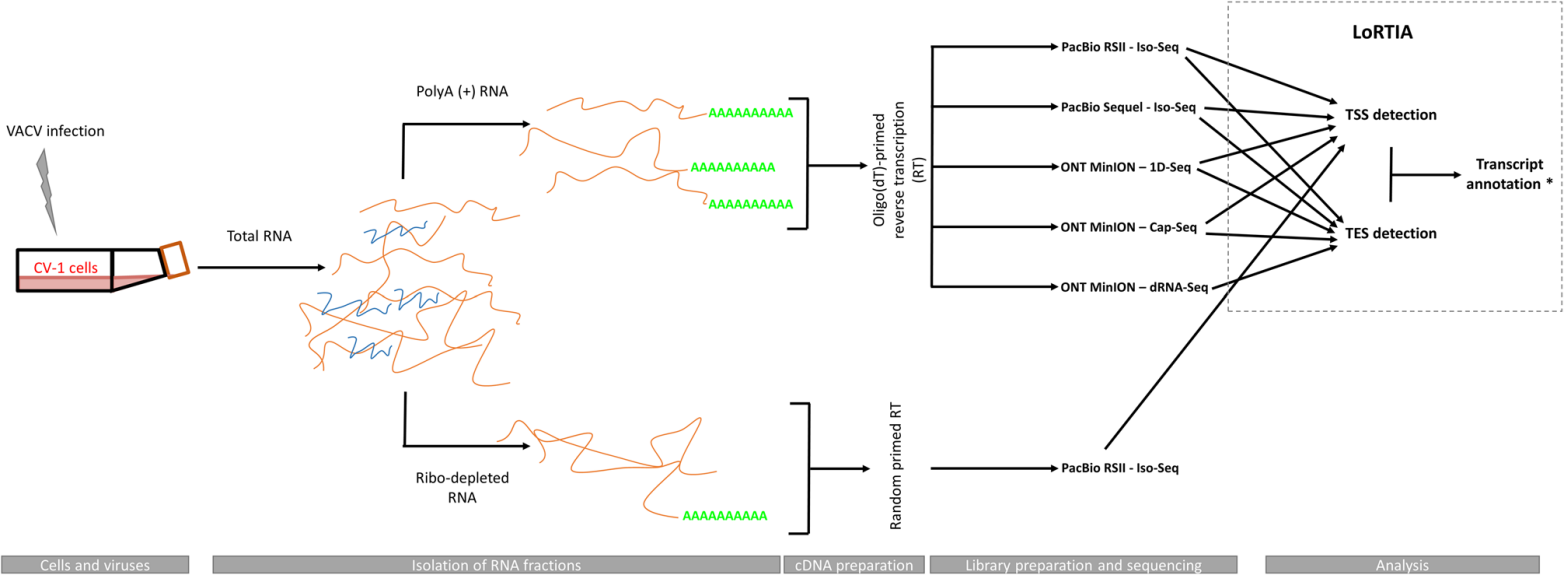
Draft workflow of the study. Infection, sequencing, and data integration strategy for transcript annotation.

In this study, we performed polyadenylation sequencing techniques for the investigation of the VACV transcriptome. We used PacBio isoform sequencing (Iso-Seq) template preparation protocol for the RSII and Sequel platforms, and the ONT MinION device to sequence cDNAs and native RNAs. For ONT sequencing, we used the company’s own library preparation approach (1D-Seq), or the Teloprime Cap-selection protocol from Lexogen, which was adapted for the MinION sequencer. The obtained datasets were then used to define and validate viral TSS and TES coordinates using the LoRTIA pipeline (https://github.com/zsolt-balazs/LoRTIA) that was developed by our research group^36^. The workflow for library preparation and analysis is shown in Fig. 1.

PCR amplification was used in all our sequencing techniques with the exception of dRNA sequencing where no amplification occurred. Direct RNA sequencing offers an alternative to cDNA sequencing because it lacks the recoding and amplification biases inherent in reverse transcription (RT) and PCR-based methods. We also used random primer-based RT for some ONT sequencing analyses. All methods generated high-coverage LRS data and determined both TSSs and TESs of viral transcripts with base-pair precision (see below). LRS can be used to detect individual RNA molecules and identify alternatively transcribed and processed RNAs, polycistronic transcripts, and transcriptional overlaps. Despite this straightforward utility, identification of transcript ends (especially of TSSs) was extremely challenging due to the tremendous complexity of the VACV transcriptome. The amplified SMRT Iso-Seq technique utilizes a switching mechanism at the 5′-end of the RNA template, thereby generating full-length cDNAs^37^.

An advantageous feature of the PacBio technique is that any error that arises can be easily corrected due to its high consensus accuracy^38^. In this study, we used a workflow and pipeline for transcriptome profiling of long-read RNA sequencing data that was developed in our laboratory^30,32,39^. In total, about 1,115,000 reads of inserts (ROIs) were generated using the PacBio platform (Supplementary Table S1). In addition to PATs, ROIs can potentially be produced non-specifically from A-rich regions of the RNA molecules^36^. Thus, we excluded these products from further analysis using the LoRTIA toolkit. Non-specific binding of the adapter and PCR primer sequences to cDNAs can also lead to false positives and we excluded these artefacts using bioinformatic filtering. We also used the LoRTIA toolkit to confirm that the 5′- and 3′-termini of the sequencing reads represented real TSSs and TESs, respectively. When two or more sequencing reads with the same TSS contained different TESs, they were considered independent ROIs. Similarly, ROIs with different PAT lengths were regarded as independent. We also accepted those TSSs that have been described by others^19,40–46^.

The advantages of the ONT MinION sequencing technique over the PacBio platform include cost-effectiveness, higher read output, and the ability to read sequences within the range of 200 to 800bps, in which PacBio and the SRS techniques are less effective^47^. Although this method is hampered by high error rates, sequencing accuracy is not particularly detrimental to transcriptome analyses of well-annotated genomes, such as that of VACV^48^. In addition to the oligo(dT)-based 1D protocol from ONT, we prepared a random hexanucleotide-primed RT to detect the non-polyA(+) RNA fractions and to validate TSSs. A Cap-Seq approach was used to identify TSS positions. Together, the nanopore sequencing methods yielded approximately 535,000 viral sequencing reads.

VACV ORFs are relatively short (Supplementary Table S2) and many of them overlap with each other. Because the PacBio MagBead loading protocol selectively removes DNA fragments shorter than 1kb^49^, potential monocistronic transcripts with a short ORFs are underrepresented or missing in these datasets. Nonetheless, in analyses of very long polycistronic and complex transcripts, PacBio has better sequencing precision than ONT. Thus, the combined use of these LRS methods eliminates the shortcomings of both. Native RNA sequencing can circumvent spurious transcription reads that are generated in PCR and RT reactions by false priming, template switching, or second-strand synthesis. The major disadvantage of dRNA sequencing is that a few bases are always missing from the 5′-ends of transcripts, and in many cases also from the 3′-ends^50^. Under these sequencing conditions, it is assumed that the lacking 5′-end nucleotides are the consequence of perturbed base calling due to premature release of the mRNA from the motor protein, which hasten the progress of RNA molecules across pores. The frequent absence of PATs in sequencing reads is explained by the miscalling of adapter nucleotides that are ligated downstream of PATs on the RNA molecule, thus muddling of the raw signal of the downstream ‘A’ homopolymer. The present dRNA-Seq analysis returned a total of approximately 195,000 reads.

This study demonstrates an extremely complex transcription pattern along the entire viral genome. Our sequencing approaches detected transcriptional activity at every nucleotide of the VACV genome. The number of annotated ORFs vary between 201 and 263^3,51^ depending on the strain and study. We identified 218 ORFs in the WR strain (GenBank accession number is LT966077.1) using in silico* methods*. 184 out of 218 ORFs were expressed either as exclusively monocistronic or as both mono- and polycistronic RNA molecules. We detected 8,191 unique putative transcripts using the LoRTIA pipeline (Supplementary Table S3a). When we applied a criterion of accepting only those transcripts which were identified by two different techniques and/or in three independent experiments, this number decreased to 1480 transcripts (Supplementary Table S3b), including potential mRNAs, non-coding transcripts, or RNA isoforms. Transcripts annotated by LoRTIA represented 175 ORFs (Supplementary Table S3b, c). Altogether 99.32% of TSSs, and 98.62% of TES positions of the annotated 1,480 transcripts were reobtained by Guppy basecalling (Supplementary Table S3b). Our dataset contains full-length transcripts for about 20 additional ORFs (Supplementary Table S3d), but no full-length reads were detected for about 25 ORFs. These regions contain several transcriptional reads without exact TSS and/or TES positions.

**Figure 2:**
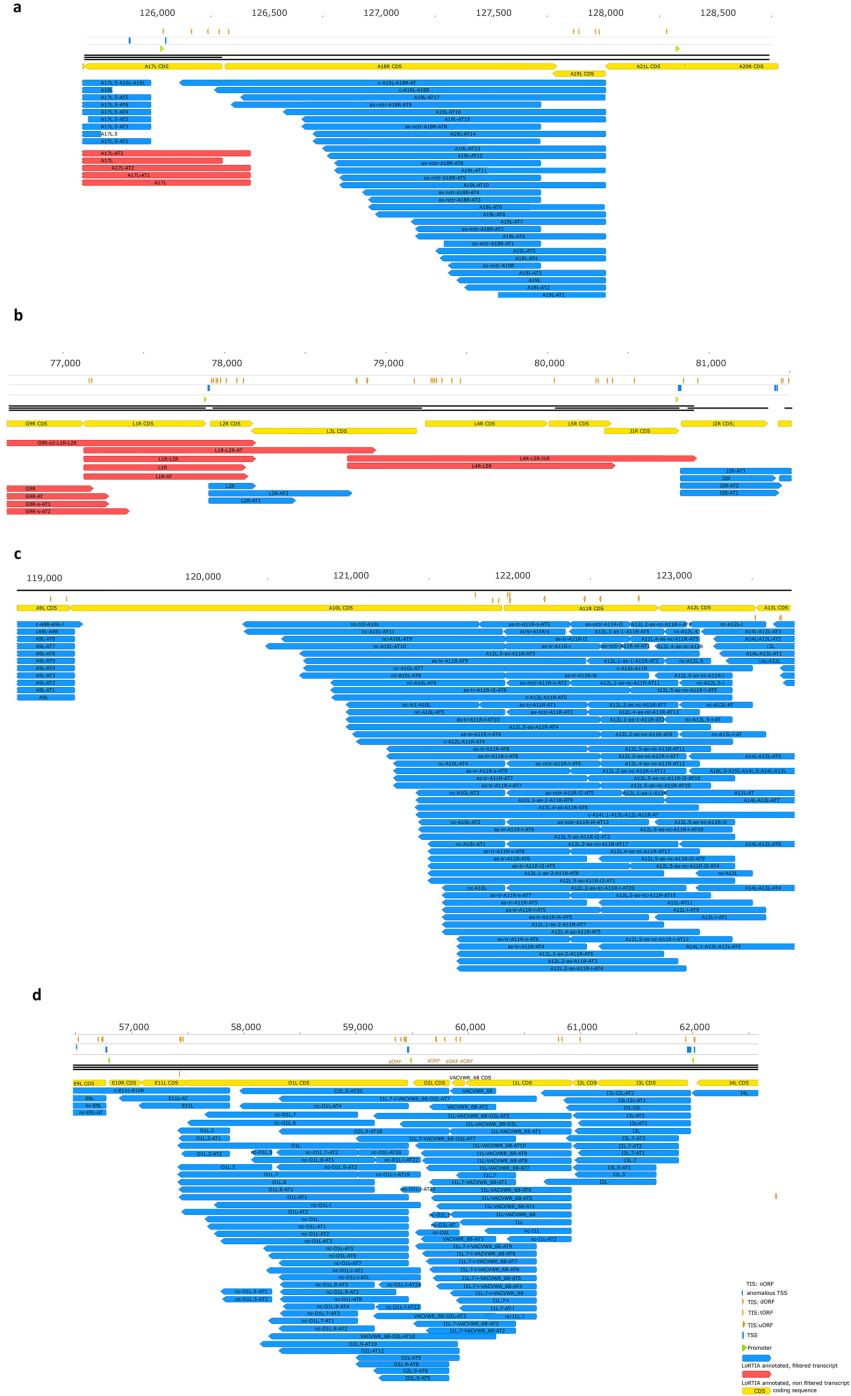
Complexity of VACV ‘chaotic regions’. (**a**) high expression of antisense transcripts within the A18R locus; (**b**) transcription start and end sites within the open reading frames (ORFs) of L genes; (**c**) extremely high expression levels of antisense RNAs and other non-coding transcripts at the A10L-A12L region; (**d**) enormous numbers of non-coding transcripts and transcript isoforms within the O1L–I3L genomic segment. All transcripts were identified using the LoRTIA toolkit.

We introduce the concepts of ‘regular’ and ‘chaotic’ genomic regions. At the ‘regular’ genomic segments viral genes produce transcript with relatively exact TSSs and TESs (Supplementary Fig. S1). However, at the ‘chaotic’ genomic loci the transcripts exhibit high heterogeneity with respect to the exact positions of TSSs and TESs (Fig. 2). Transcripts with highly diverse transcription initiation and termination sites are encoded by the I and L genes (such as O1L, O2L, I1L, I2L, I3L, A10L, A11R, A12L, A18R genes) at particularly the late time points of the infection. This phenomenon creates considerable difficulty in identifying individual transcripts.

### Putative protein-coding genes

Herein, we identified (a) genes in new genomic positions, (b) embedded genes (5′-truncted in-frame ORFs within larger canonical ORFs), and (c) short upstream ORFs (uORFs, preceding the main ORFs). We considered the most abundant transcript isoform specified by a given gene as the canonical (main) transcript. (Note S1). The novel ORFs and transcripts were named according to the common (Copenhagen) HindIII fragment letter/number-based nomenclature^45^. All VACV transcripts (identified by the LoRTIA pipeline) are presented in a Geneious file available at FigShare (Supplementary Note S2).

#### Genes at novel genomic positions

We detected two novel putative mRNAs with short ORFs (both 51bps) that are located in the intergenic genomic regions (B25.5R/C19.5L, C9.5L) of two divergently oriented genes. The TSS positions of these transcripts have previously been reported^14,19^ (Supplementary Table S4). Our analysis showed that these ORFs are expressed both alone in separate transcripts and as upstream ORFs (uORFs) located 5′ of canonical ORFs in longer transcripts.

#### Embedded genes

**Figure 3:**
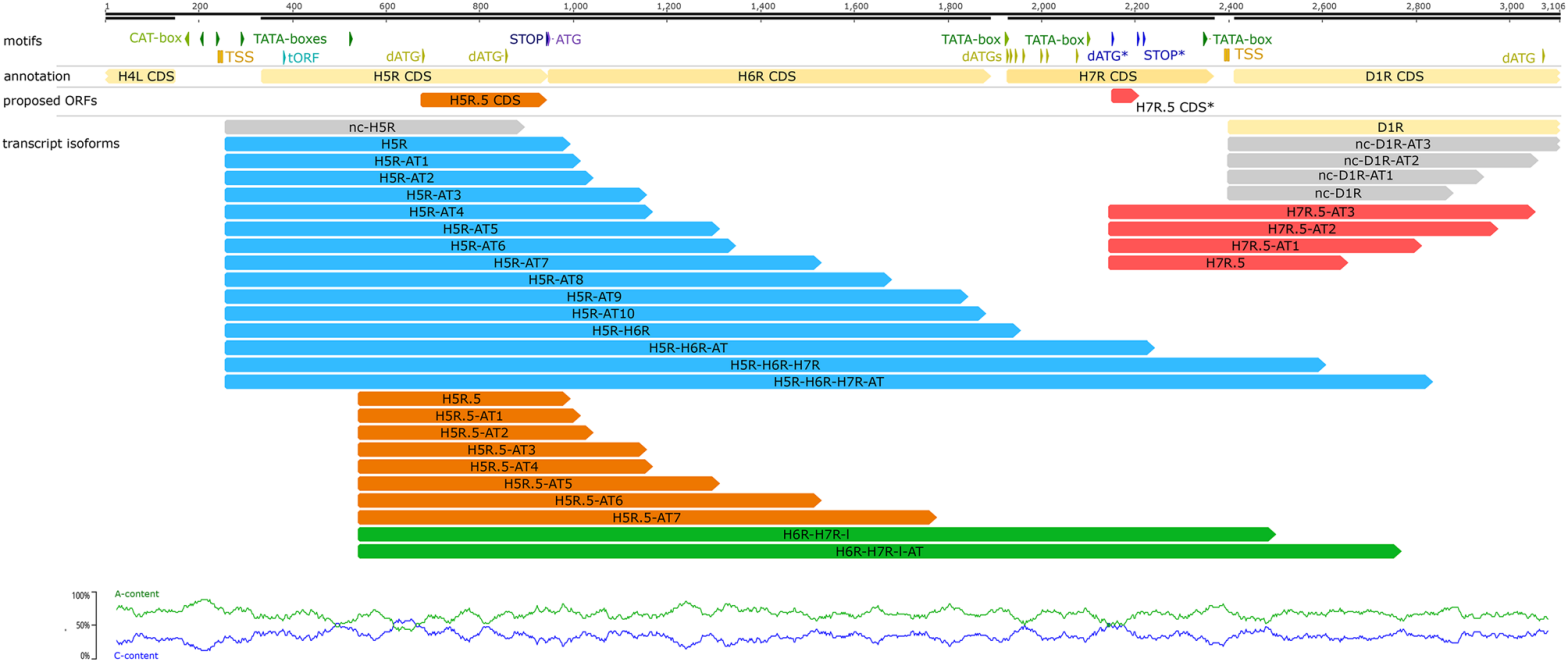
Schematic of two examples of the identified putative embedded genes. We detected a number of 5′-truncated transcripts containing short in-frame ORFs. This figure shows transcripts from H5R.5 and H7R.5 ORF regions.

A characteristic feature of the VACV genome is that it contains several short in-frame ORFs, called embedded genes, many of which are translated^12,40^. Using full-length RNA sequencing we identified 49 novel putative embedded genes with 5′-truncated in-frame ORFs, which were shorter than the in silico annotated canonical ORFs (Fig. 3). This study demonstrated that these putative genes specify more than 300 transcript isoforms. We initially assumed that the TISs of these internal ORFs were the closest in-frame ATGs to canonical host ORFs. However, this is not necessarily the case, a large number of TISs were detected by others using ribosome profiling^12^. We identified 22 unique promoters associated with these TISs using our in-house scripts but we detected only six transcripts that contain in-frame ORFs (Supplementary Table S5).

#### Upstream ORFs

Upstream ORF-containing transcripts are considered to play a regulatory role in eukaryotic gene expression^52^. Translation of uORFs typically inhibit downstream expression of canonical ORFs. The 5′-UTR of uORF-containing transcripts are reportedly ≥ 75 bp in length^52^. Several in silico and experimental studies have shown that 40–50% of human and mouse mRNAs contain at least one uORF^53,54^. We applied strict criteria for filtering data obtained by the LoRTIA toolkit and thereby revealed that 25 previously annotated VACV genes express longer TSS variants than the canonical transcripts. On average, the 5′-UTRs of the main transcript isoforms were 101 bp (Supplementary Fig. S2), whereas the longer variants were 258 bp long (Supplementary Table S6). Most of these transcript isoforms (64%) contain at least one ORF preceding the main ORF with an average 5′-UTR length of 331 nts. We set the minimal size limit of these uORFs to 9 nts comprising at least one triplet between the putative start and stop codons, as described by Scholz and colleagues^55^. We also set a maximal ORF length of 90 nts and considered transcripts with longer upstream ORFs as bicistronic. Eleven VACV transcripts with 5′-UTR variants were found to contain multiple uORFs. G5.5R was the only gene in which the longer transcript isoform contained a single uORF. The VACV transcriptome is distinguished by a high number of TSS and TES positions and.

#### Non-coding transcripts

In this work, we identified 356 novel putative long non-coding RNAs (lncRNAs; ≥ 200 bps) using the LoRTIA toolkit (Supplementary Table S3b). We also identified 13 potential short ncRNAs (sncRNAs; ≤ 200 bps) with lengths varying between 121 and 193 bps.

#### Antisense RNAs

**Figure 4:**
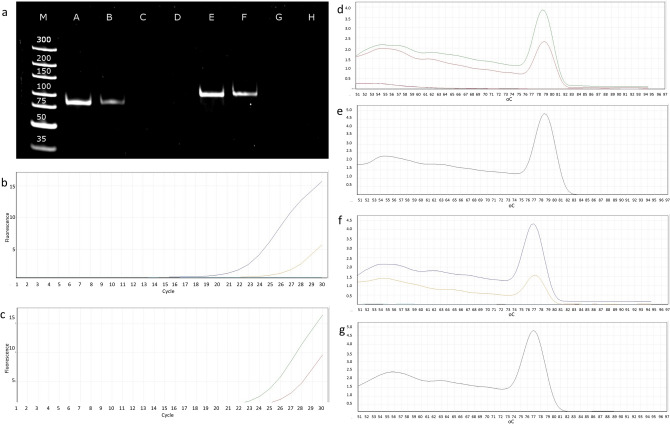
Detection of antisense RNA expression from the complementary DNA strands of two VACV genes using qRT-PCR. (**a**) Polymerase chain reaction (PCR) products of the A11R transcript and its antisense partner. Abbreviations: M: molecular weight marker; A: A11R antisense RNA; B: A11R mRNA; C: A11R antisense RNA—NO-RT control; D: A11R mRNA—NO-RT control; E: A18R antisense RNA F: A18R mRNA; G: A18R antisense RNA—NO-RT control; H: A18R mRNA—NO-RT control. (**b**) amplification curves of the *A18R* gene and its antisense transcripts. (**c**) Amplification curves of the A18R gene and its antisense transcripts. (**d**–**g**) Melting curves are shown to demonstrate the specificity of the amplification. The curves of transcripts cross the threshold line within 19.5–23.4 cycles, whereas the curves of NO-RT controls remain flat. (**d**) A11R mRNA and antisense RNA. (**e**) A11R DNA control. (**f**) A18R mRNA and antisense RNA (**g**) A18R DNA control.

With few exceptions, we detected transcriptional activity from both DNA strands along the entire viral genome. The ratio between the mRNAs and antisense RNAs (asRNAs) varied between genomic locations. After applying the strict criteria to the LoRTIA tool, exactly 100 antisense transcripts were located at the genomic loci A5R (a single transcript), A11R (89 transcripts), and A18R (10 transcripts) (Fig. 4). The A11R antisense transcripts are various combinations of six TSS and 40 TES positions, whereas antisense A18R transcripts have the same TSSs but vary in their termination sites. Some of these transcripts contain small ORFs, which might indicate coding potentials (Note S2). In most cases, the level of asRNA expression was relative low, that is why the LoRTIA tool was unable to identify these molecules.

#### Intragenic non-coding transcripts

After applying our strict filtering criteria, we identified 260 3′-truncated transcripts from the LoRTIA dataset. We considered these as non-coding transcripts because they lacked in-frame stop codons. However, we cannot exclude the possibility that they utilize an out-of-frame ORF for protein coding. These transcripts were detected to be expressed from 41 viral genes (36.15% of them are located within the O1L region). The A37R (8.85%) and A10L (5.38%) regions and the F12L-F13L gene cluster (11.15%) also expressed a substantial number of 3′-truncated RNAs. Altogether 62% of the 3′-truncated ncRNAs are located within five VACV ORFs (Supplementary Table S3b).

#### Replication-associated transcripts

The VACV genome contains multiple replication origins (Oris)^56^ located near the genomic termini. Oris are overlapped by many low-abundance ncRNAs; however, due to their large variation in length, their TSSs and TESs were not identified by either LoRTIA or other methods. We demonstrate that these RNAs are expressed from 4 h post infection (p.i.), and they may play similar roles as the replication-associated RNAs of other herpesviruses^57^.

### Novel mono-, bi- and polycistronic RNAs

In ‘regular’ genomic regions we detected 135 monocistronic coding transcripts of previously annotated ORFs using the LoRTIA software suit, and 12 additional mRNAs that did not meet the LoRTIA criteria due to their low abundance (Supplementary Table S7). Similar to other viruses and prokaryotes, but unlike the eukaryotic organisms, VACV reportedly express bi- and polycistronic transcripts^58–60^. Our study detected altogether 43 bicistronic, 137 tricistronic, 92 tetracistronic, 15 pentacistronic, and 5 hexacistronic RNA molecules (Supplementary Table S3b, c).

### Complex transcripts

We term complex RNAs (cxRNAs) those multigenic transcripts which contain at least two ORFs in opposite orientations. This study detected 30 cxRNA molecules (Supplementary Table S3b, c, d, S8, Note S2) encoded by nine genomic segments. Six of these are TES isoforms of the A12L–A11R complex transcript, five cxRNAs are the combinations of TSSs and TESs within the A14L-A13L-A12L-A11R region, and four transcripts are alternative TES variants of VACVWR_161-A38L RNA. Three additional complex transcripts were excluded by LoRTIA analyses due to the low coverage at this genomic region. Since most VACV genes stand in tandem orientation with each other, and only a few convergently and divergently positioned gene pairs are present, the number cxRNAs is low compared to herpesviruses. Two cxRNAs (c-A21L-A20R, c-G3L-G2R) were considered non-coding because their upstream genes stand in an antisense orientation on the transcript. The remaining complex transcripts were categorized as mRNAs, because their first ORFs stand in the sense direction. We detected cxRNAs with convergently-oriented genes at E10R-E11L and A8R-A9L genomic regions. Fully overlapping convergent cxRNAs were also detected in the E10R-E11L region (Supplementary Fig. S3).

### LRS reveals highly complex isoform heterogeneity

Transcript isoforms include 5′- and 3′-UTR length variants of RNA molecules. Using tag-based RNA-Seq methods with SOLiD and Illumina sequencing platforms, Yang and co-workers determined 5′- and 3′-ends of VACV E mRNAs with high precision^14,19^ and also mapped terminal sequences of I and L transcripts^40^. These authors also described the expression profiles of I and L RNA-products^16^. These studies identified several hundreds of TSSs and PASs; but they were unable to ascertain the precise 3′-ends of RNAs due to the high transcriptional complexity^14^.

#### Transcription start sites

**Figure 5:**
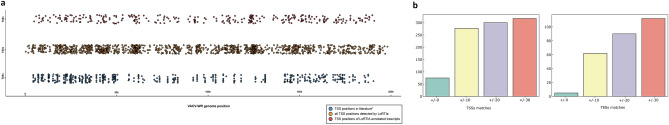
Jitter plot visualization of transcriptional start site distribution across the VACV genome. (**a**) Distribution of TSSs of our annotated transcripts are shown at the top (red dots); the TSSs detected using LoRTIA are depicted in the middle (orange). TSS positions described by others^14,15,19,40^ are located at the bottom of the figure (blue). (**b**) TSS and TES positions detected by LoRTIA share the TSS and TES positions obtained from other studies (Supplementary Table S9) with different frequency. Bar charts represent the fall of TSS and TES positions within a ± 10–20–30 sliding window.

We annotated 1073 TSSs of VACV transcripts, 987 of which have not previously been detected (Supplementary Table S9). Eighteen percent of TSSs matched with base-pair precision to those described by Yang and colleagues^14,15,19,40^. This value increased to 70% when we allowed a ± 10-nt, and to 93% once we allowed a ± 30-nt precision interval for the location of TSSs (Fig. 5a). Altogether 898 TSSs were detected in ‘regular’ regions. The previously published TSS positions are depicted in FigShare (Supplementary Note S2).

#### Transcription end sites

The TESs of early transcripts were increasingly heterogenic at late time points of infection, potentially because transcription termination signal recognition depends on different factors in early- and PR phases^12,14,15,19^. Due to the extremely high TES diversity (Supplementary Fig. S4), we analysed their positions within a ± 30-nt interval. We found that 24% of our annotated TESs matched the positions of the polyadenylation sites described by Yang et al.^15^ (Fig. 5b). Moreover, most genes express multiple transcript isoforms (median = 4, mean = 6.95), up to 30 in extreme cases, such as in regions.N1L, O2L, I1L, and A12L (Supplementary Table S3b; Supplementary Fig. S5). Moreover, no TSS positions belonging to 30 annotated ORFs, particularly in the A8R-A17L region, have been annotated in our and others’ studies (Note S3).

### Cis-regulatory elements

**Figure 6:**
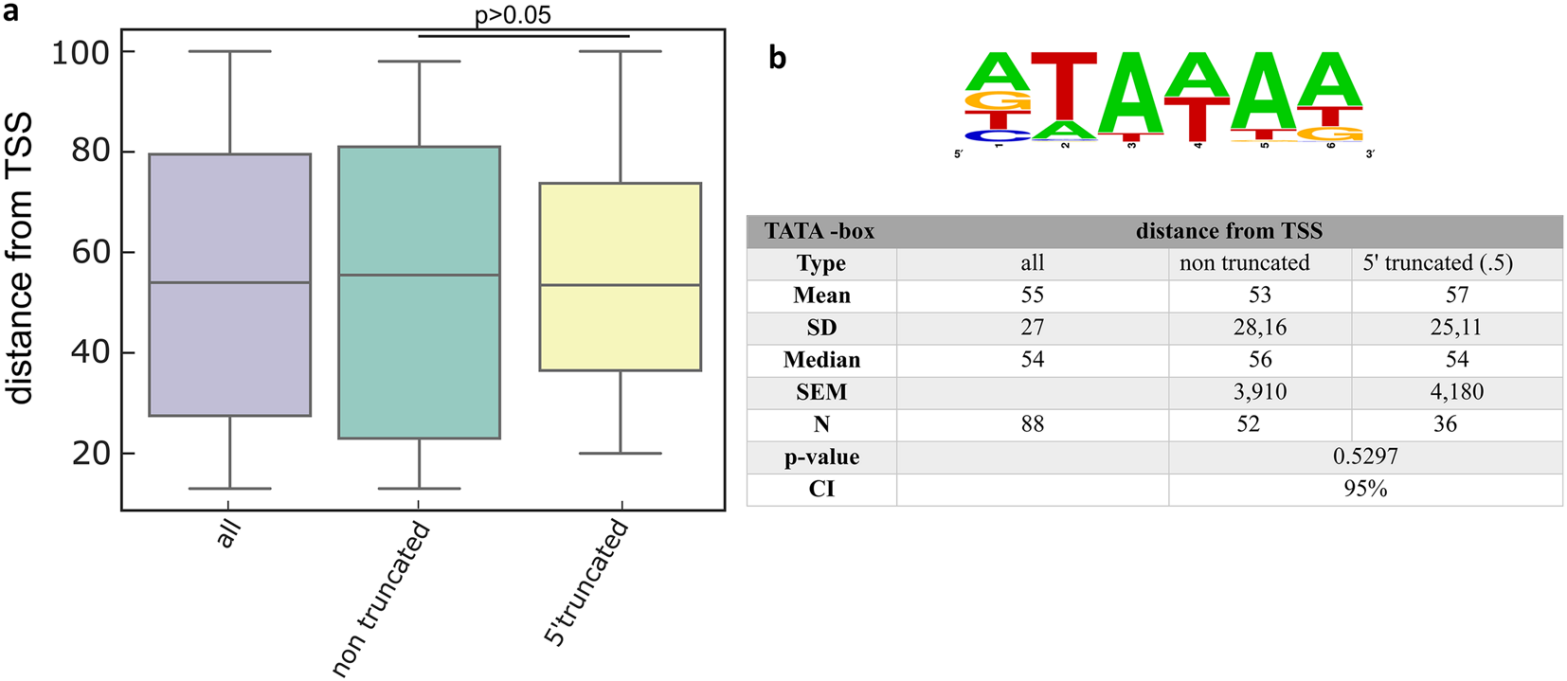
In silico analysis of promoter elements. (**a**) The bar chart shows average distances between the TATA boxes and TSSs. (**b**) The WebLogo shows the consensus sequence of the identified TATA boxes.

Yang and colleagues described a 15-nucleotide consensus promoter core motif upstream of E genes, but these sequences were also present at other genomic locations. We identified this motif at nine novel positions upstream of (C9.5L, F4L, F7L, J3R, A15R, VACWR_161, and A51R) and inside (A36R and VACWR_169) ORFs. Cis-regulatory elements have not previously been annotated within these regions (Note S2). However, these core motifs are not present in the chaotic genomic regions. Additionally, most TESs of novel transcripts are located about 50 nucleotides downstream of UUUUUNU termination signals, as described by Yang et al.^19^. Those TESs which were not preceded by this motif might use an alternative mechanism for polyadenylation-site selection. Matching TSSs with TESs can only be carried out using LRS techniques, which are therefore crucial for the transcriptome analysis, especially in VACV which exhibits a very complex gene expression profile. We found that in certain DNA segments of ‘chaotic’ regions almost all base positions can function as TESs. Transcription initiation was also found to be stochastic at these regions. Accordingly, our analysis revealed that VACV genes express transcripts with multiple shared TSSs (Fig. 2a–c, Supplementary Fig. S4a) and TES positions (Fig. 2c, d, Supplementary Fig. S3b). Distributions of TSSs and TESs along the VACV genome are presented in Supplementary Fig. S6. We identified TSSs for 90% of the promoters described by Yang et al., (2010), with the exception of G2R, J6R, A18R, A20R, and five hypothetical genes located in the repeat region of the VACV genome. We used in silico promoter predictions to detect potential TATA- and CAAT-box consensus sequences of the present TSSs. We analysed 146 TSSs without previously annotated promoters and found TATA-boxes in 88 (Fig. 6, Supplementary Table S10), and CAAT-boxes in 5 of them (Supplementary Table S10). The median distance between a TSS and a putative TATA-box was 54 nts. Yet, no significant differences in promoter–TSS distances were identified between 5′-truncated and canonical transcript isoforms, suggesting that these cis-regulatory structures were similar in these two types of VACV genes (Fig. 6).

### Transcriptional overlaps

Using multiplatform analyses, we revealed an extremely complex meshwork of transcriptional overlaps, which were expressed by all viral genes. Cumulatively, 154 tandem, 32 divergent, and 32 convergent gene pairs were found in the VACV genome, with 21 overlapping ORFs between tandem genes, 13 between convergent gene pairs, and 5 between divergent gene pairs. Moreover, closely-spaced tandem genes can form partial and full parallel overlaps. We also detected 106 parallel transcriptional overlaps between tandem genes (Supplementary Table S11). Seventeen of the 32 convergent gene pairs formed UTR-UTR overlaps (Supplementary Table S11), whereas only 15 gene pairs formed UTR-ORF overlaps. Twelve of the 16 divergently-oriented gene pairs were also found to form transcriptional overlaps with each other (Supplementary Table S11). Polycistronic and complex transcripts are also the results of transcriptional overlaps (Supplementary Table S12).

## Discussion

Short-read sequencing has become a standard technique for the characterization of transcriptomes^61,62^. Yet comprehensive annotation of transcripts from SRS data remains challenging^63^. Auxiliary methods, such as Northern-blotting and rapid amplification of cDNA ends analyses are too laborious for investigations of large transcriptomes. Long-read sequencing allows the determination of full-length transcripts in single reads without computational inference, and thus distinguishes between transcript isoforms, polycistronic RNAs, and transcript overlaps with ease^64^. Previous studies using Illumina sequencing have detected large numbers of TSSs, TESs, and TISs of VAVC transcripts^12,15,19^. However, these analyses have not identified transcripts because SRS techniques are unable to match TSSs and TESs.

In this study, we employed two LRS platforms (PacBio and ONT) and sequenced cDNAs and native RNAs using various library preparation methods for surveying the temporal transcriptional activities of VACV genes. We developed pipelines for profiling long-read RNA sequencing data. In actuality, RNA molecules expressed by the virus may be much more numerous than we annotated. This may be due to a high heterogeneity of transcript ends, especially in ‘chaotic’ regions in the viral genome, which are difficult to analyse by bioinformatic techniques. The intermediate and late transcripts are particularly polymorphic in length, but the variation in TSS and TES positions of early transcripts is also increased at late stages of infection. Generally, canonical transcripts are significantly overrepresented among isoforms encoded in ‘regular’ genomic regions. Apart from canonical transcripts, VACV genes typically encompass shorter genes with N-terminal truncated ORFs, which may encode shorter polypeptides, possibly with altered effector functions. Moreover, longer transcript variants often incorporate uORFs, which may regulate the expression of downstream genes^32^.

The genomic organization of poxviruses differs from that of herpesviruses in many ways. Poxvirus E genes are gathered at the genome termini, whereas I and L genes are located in central parts of the DNA molecule. In addition, unlike herpesviruses, VACV DNA encodes numerous TSS and TES isoforms. While prototype herpesvirus transcripts are organized into tandem gene clusters generating overlapping transcripts with co-terminal RNA molecules, adjacent pox genes produce large numbers of alternative TESs. In VACV, the heterogeneity of TESs exceeds that of TSSs, whereas the opposite is the case in herpesviruses. The general presence of within-ORF TSSs is also unique to VACV. Yang and colleagues, using ribosome profiling assays, have also identified several downstream methionine residues and mapped these within main ORFs^12^.

We detected numerous TSSs upstream of TISs, thereby providing transcriptional evidence for the existence of potential truncated proteins. We also detected long 5′-UTR isoforms containing uORFs in most VACV genes, providing a potential source of micro peptides with regulatory functions, as demonstrated previously in Kaposi’s sarcoma virus^65^. The observed transcript diversity of VACV may, however, represent transcriptional noise from cryptic promoters and/or error-prone transcriptional machinery. Moreover, the majority of transcripts contain multiple translationally active ORFs, and most isoforms contain unique combinations of ORFs and uORFs, suggesting that this transcriptional diversity is functional. Hence, further studies are needed to demonstrate the biological significance of these RNA molecules.

We provide evidence that poly(A) signals are frequently transmitted by RNA polymerase without downstream cleavage of transcripts. This process results in the production of polycistronic RNAs from tandem genes and asRNAs from convergent gene pairs. The ensuing stochastic transcription initiation and termination in poxviruses represents a unique gene expression system, which has not yet been described in other well-characterized organisms. No previous studies show translation of polycistronic RNA molecules from downstream genes in poxviruses. To explain this phenomenon and read-through transcription in herpesviruses^66,67^, we hypothesize that transcriptional overlaps are sites of transcriptional interference. These interactions have been suggested to be a part of a genome-wide regulatory system, coined transcriptional interference networks (TINs)^68^. We report sources of potential error in RT, PCR, and bioinformatic techniques, and propose strategies for addressing these. In particular, we applied very strict criteria for accepting sequencing reads as transcripts. However, a large fraction of excluded reads could represent existing transcripts.

## Methods

### Cells and viruses

CV-1 African green monkey kidney fibroblast cells were obtained from the American Type Culture Collection. Cells were plated at a density of 2 × 10^6^ cells per 150-cm^2^ tissue culture flask and were cultured in RPMI 1640 medium (Sigma-Aldrich) supplemented with 10% foetal bovine serum and antibiotic–antimycotic solution (Sigma-Aldrich). CV-1 cells were incubated at 37 °C in a humidified atmosphere containing 5% CO_2_ until confluent. Subsequently, about 2.6 × 10^7^ cells were rinsed with serum free RPMI medium immediately prior to infection with the vaccinia virus WR strain diluted in serum free RPMI medium.

### Infection

Cells were infected with 3-ml aliquots of VACV at a multiplicity of infection of 10/cell, and were incubated at 37 °C in an atmosphere containing 5% CO_2_ for 1 h with brief agitation at 10-min intervals to redistribute virions. Three-mL aliquots of complete growth medium (RPMI + 10% FBS) were then added to tissue culture flasks and the cells were incubated at 37 °C for 1, 2, 3, 4, 6, 8, 12, and 16 h in a humidified atmosphere containing 5% CO_2_. Following incubation, media were removed and cells were rinsed with serum free RPMI 1,640 medium and were subjected to three cycles of freeze–thawing. Cells were finally scraped into 2-ml aliquots of PBS and were stored at − 80 °C until use.

### RNA purification

Total RNA was extracted from infected cells at various stages of viral infection using Macherey–Nagel RNA kit according to the manufacturer’s instructions. Polyadenylated RNA fractions were isolated from total RNA samples using Oligotex mRNA Mini Kit (Qiagen) following the Spin-Column Protocol. We used Ribo-Zero Magnetic Kit H/M/R (Illumina) to remove rRNAs from total RNA samples for analyses of non-polyadenylated RNAs.

### PacBio RSII and sequel sequencing

#### cDNA synthesis

Copy DNAs were generated from polyA(+) RNA fractions using SMARTer PCR cDNA Synthesis Kits (Clontech) according to ‘PacBio Isoform Sequencing (Iso-Seq) using Clontech SMARTer PCR cDNA Synthesis Kit and No Size Selection’ protocols. Samples from different infection time points (1-, 4-, 8-, and 12-h p.i.) were mixed for RSII library preparation, whereas 1-, 2-, 3-, 4-, 6-, and 8-h p.i. samples were mixed for Sequel sequencing. Samples of cDNA were prepared from rRNA-depleted RNA mixtures from 1-, 4-, 8-, and 12-h time points using modified random hexamer primers (Supplementary Table S13) instead of using the oligo(d)T-containing primer provided in the SMARTer Kit. Samples were used for SMRTbell template preparation using the PacBio DNA Template Prep Kit 1.0.

#### SMRTbell library preparation and sequencing

The detailed version of the template preparation protocol is described in our earlier publication^69^. Briefly, primer annealing and polymerase binding reactions were performed using the DNA Sequencing Reagent Kit 4.0 v2 with DNA Polymerase P6 for the RSII platform, whereas the Sequel Sequencing Kit 2.1 and Sequel DNA Polymerase 2.0 were applied for sequencing on the Sequel. Polymerase-template complexes were bound to magbeads prior to loading into the PacBio instrument. Reactions were then performed using The PacBio’s MagBead Kit (v2). Finally, 240- or 600-min movies were captured using the RSII and Sequel machines, respectively. One movie was recorded for each SMRTcell.

### ONT MinION platform–cDNA sequencing

#### 1D cDNA library preparation

PolyA(+) RNA fractions were used for cDNA sequencing on the MinION device. RNAs from different infection time points were converted to cDNAs according to the ONT 1D strand-switching cDNA ligation protocol (Version: SSE_9011_v108_revS_18Oct2016). An RNA mixture containing equal amounts of RNA from 1-, 2-, 3-, 4-, 6-, 8-, 12-, and 16-h p.i. were prepared for sequencing. Libraries were generated using the above mentioned 1D ligation kit and protocol, the Ligation Sequencing 1D kit (SQK-LSK108, ONT), and the NEBNext End repair/dA-tailing Module, NEB Blunt/TA Ligase Master Mix (New England Biolabs) according to the manufacturer’s instructions. Briefly, polyA(+)-selected RNAs were converted to cDNAs using Poly(T)-containing anchored primers [(VN)T20; Bio Basic, Canada], dNTPs (10 mM, Thermo Scientific), SuperScript IV Reverse Transcriptase Kit (Life Technologies), RNaseOUT (Life Technologies), and strand-switching oligonucleotides with three O-methyl-guanine RNA bases (PCR_Sw_mod_3G; Bio Basic, Canada). The resulting double-stranded cDNAs were amplified by PCR using KAPA HiFi DNA Polymerase (Kapa Biosystems), Ligation Sequencing Kit Primer Mix (from the ONT 1D Kit), and a Veriti Thermal Cycler (Applied Biosystems). The NEBNext End repair/dA-tailing Module was used to blunt and phosphorylate cDNA ends, and the NEB Blunt/TA Ligase Master Mix was used for adapter (supplied in the 1D kit) ligation.

Size selection: PCR products from mixed RNA samples were size-selected manually and were then run on Ultrapure Agarose gels (Thermo Fischer Scientific). Subsequently, fragments of greater than 500-bp were isolated using Zymoclean Large Fragment DNA Recovery Kits (Zymo Research).

Barcoding: Individually sequenced cDNAs were barcoded using a combination of the following ONT protocols: the 1D protocol was used until the first end-preparation step, then we switched to the 1D PCR barcoding (96) genomic DNA (SQK-LSK108) protocol (version: PBGE96_9015_v108_revS_18Oct2016, updated 25/10/2017), followed by the barcode ligation step using the ONT PCR Barcoding Kit 96 (EXP-PBC096). Barcode adapters were ligated to end-prepped samples using Blunt/TA Ligase Master Mix.

#### Library preparation from cap-selected samples

For more accurate definitions of TSSs of full-length RNA molecules, we followed the 5′-Cap-specific cDNA generation protocol combined with the ONT 1D-seq library preparation method. We produced cDNAs from a mixture of total RNA samples (1-, 2-, 3-, 4-, 6-, 8-, 12-,16-h p.i.) using TeloPrime Full-Length cDNA Amplification Kits (Lexogen). Amplified PolyA- and Cap-selected samples were then used for library preparation following the ONT 1D strand-switching cDNA ligation method (ONT Ligation Sequencing 1D kit). Samples were then end-repaired (NEBNext End repair/dA-tailing Module) and ligated to ONT 1D adapters (NEB Blunt/TA Ligase Master Mix).

#### Sequencing on the MinION device

ONT 1D-cDNA and Cap-selected libraries were loaded onto three and two ONT R9.4 SpotON Flow Cells for sequencing, respectively. Sequencing runs were performed using MinKNOW.

### ONT MinION platform–dRNA sequencing

To avoid probable amplification-based biases, we used the ONT PCR-free direct RNA (dRNA) sequencing protocol (Version: DRS_9026_v1_revM_15Dec2016). A mixture containing PolyA(+) RNAs from eight time points (1-, 2-, 3-, 4-, 6-, 8-, 12-, 16-h p.i.) was used to generate the library. RNA samples, the oligo(dT)-containing adapter (ONT Direct RNA Sequencing Kit; SQK-RNA001), and T4 DNA ligase (2 M U/mL; New England BioLabs) were mixed and incubated for 10 min, and first-strand cDNAs were then prepared using SuperScript III Reverse Transcriptase enzyme (Life Technologies) according to the dRNA protocol. The RMX sequencing adapter was ligated to the samples with NEBNext Quick Ligation Reaction Buffer and T4 DNA ligase. The dRNA library was run on a R9.4 SpotON Flow Cell. Runs were performed using MinKNOW.

### Purification of libraries

Samples were purified using Agencourt AMPure XP magnetic beads (Beckman Coulter) after each enzymatic steps during PacBio and ONT library preparation. Beads were handled before use with RNaseOUT (40 U/μL; 2U enzyme/1μL bead) for dRNA libraries.

### Data analysis and visualization

#### Generation of consensus sequences from the PacBio dataset

ROI reads were created from the RSII raw data using the RS_ReadsOfInsert protocol (SMRT Analysis v2.3.0) with the following settings: Minimum Full Passes = 1, Minimum Predicted Accuracy = 90, Minimum Length of Reads of Insert = 1, Maximum Length of Reads of Insert = No Limit. ROIs from the Sequel dataset were generated using SMRT Link5.0.1.9585.

#### ONT dataset–basecalling

MinION base calling was performed using the ONT Albacore software v.2.0.1. The newest release of the ONT’s Guppy (Guppy v.3.6.0) was also used for basecalling with the aim to validate the TSS and TES positions of the annotated transcripts using a ± 10 bp window.

#### Mapping and annotation of TSS and TES positions and transcripts

PacBio ROIs and ONT raw reads were aligned to the reference genome of the virus (LT966077.1); RefSeq assembly accession: GCF_000 409795.2 [latest]) using minimap2 aligner (version 2.13) with the options -ax splice -Y -C5–cs. After applying the LoRTIA toolkit (https://github.com/zsolt-balazs/LoRTIA), the determined 5′- and 3′-ends of transcripts and detected full-length reads were mapped.

### Prediction of *cis*-regulatory sequences

Sequences at 100-nt upstream of a given TSS were extracted and an in-house script based on the GPMiner promoter prediction tool^70,71^ was used for analyses of upstream cis-regulatory sequences of novel transcripts. The general settings of the algorithm were as follows: the eukaryotic promoter database of GPMiner was used to search exact matches without gaps or substitutions. The Vaccinia early promoter motif^14,15,19^ and late promoter motif^41^ were picked and visualized using the Geneious R10 Motif Finder tool, allowing one substitution in the string. Promoter statistics were calculated using Mann–Whitney U tests with two-tailed p-values.

### Transcript naming scheme

Previously described (Copenhagen nomenclature^45^) ORFs were not renamed. Our scheme allowed for future additions of novel transcripts. Multicistronic transcripts were named for all contributed ORFs, the most upstream ORF was listed first. Non-coding transcripts are identified with the prefix “nc,” and complex transcripts carry the prefix “c.” When a non-coding transcript is antisense to the ORF for which it was named, an “as” prefix was added. Names of length isoforms, e.g., TSS and TES isoforms, end with “l” for long, “s” for short, or “AT” for alternative termination (Note S1). Isoform categories are presented in Supplementary Fig. S7.

The details for validation of specificity, quality and quantity of libraries are found in Supplementary Methods. The detailed protocols and the raw dataset were published^72^.

### Ethics declaration

All experiments using VACV were conducted under biosafety level 2. Neither human nor animal experiments were applied in this study.

## Supplementary information

Supplementary information 1

Supplementary information 2

Supplementary information 3

Supplementary information 4

## Data Availability

Relevant data are within the manuscript and its supporting information files. In addition, long-read sequencing datasets are available in European Nucleotide Archive (ENA) under the accession number PRJEB26434 and PRJEB26430. The complete map of the VACV transcriptome is available at FigShare: https://figshare.com/articles/Vaccinia_virus_transcriptome/12196275?fbclid=IwAR0WXkaqAjX4JpIrFJobklBMY15H8y40ek6_fxz1X019LzaigRdUMgSbUaY as a Geneious file.
