# Supplementary material for: Long-read assays shed new light on the transcriptome complexity of a viral pathogen
Source: Sci Rep. 2020 Aug 14;10:13822. doi: 10.1038/s41598-020-70794-5 (PMC7427789; doi:10.1038/s41598-020-70794-5)
Supplement: Supplementary file 1 — Supplementary information 1 [file 41598_2020_70794_MOESM1_ESM.docx]

1. **Long-read Assays Shed New Light on the Transcriptome Complexity of a Viral Pathogen**
2. **Dóra Tombácz^1^, István Prazsák^1^, Zsolt Csabai^1^, Norbert Moldován^1^, Béla Dénes^2^, Michael Snyder^3^, Zsolt Boldogkői^1,*^**
3. ^1^Department of Medical Biology, Faculty of Medicine, University of Szeged, Szeged, 6720, Hungary
4. ^2^Veterinary Diagnostic Directorate of the National Food Chain Safety Office, Budapest,1143, Hungary
5. ^3^Department of Genetics, School of Medicine, Stanford University, Stanford, CA 94305, USA
6. *[boldogkoi.zsolt@med.u-szeged.hu](mailto:boldogkoi.zsolt@med.u-szeged.hu)
7. **Supplementary methods**
8. **Quantitative reverse transcription PCR**

Quantitative reverse transcription-PCR (qRT-PCR; Rotor-Gene Q; Qiagen) was performed to check the specificity of cDNA products derived from Cap-Seq library preparation, and to validate the antisense expression within A11R and A18R genomic loci. Briefly, RT was performed with 20-ng total RNA samples, SuperScript IV reverse transcriptase, and oligo(dT) primers. RT products were then amplified using ABsolute qPCR SYBR Green Mix (Thermo Fisher Scientific) with gene specific primers (Table S13).

1. **Nucleic acid quality and quantity checking**

Concentrations of the reverse-transcribed and adapter-ligated RNAs were measured using a Qubit 2.0 Fluorometer (Life Technologies, Table S14). An Agilent Bioanalyzer 2100 was used for quality control of RNA samples.
