# Supplementary material for: Long-read assays shed new light on the transcriptome complexity of a viral pathogen
Source: Sci Rep. 2020 Aug 14;10:13822. doi: 10.1038/s41598-020-70794-5 (PMC7427789; doi:10.1038/s41598-020-70794-5)
Supplement: Supplementary file 2 — Supplementary information 2 [file 41598_2020_70794_MOESM2_ESM.docx]

**Long-read Assays Shed New Light on the Transcriptome Complexity of a Viral Pathogen**

**Dóra Tombácz^1^, István Prazsák^1^, Zsolt Csabai^1^, Norbert Moldován^1^, Béla Dénes^2^, Michael Snyder^3^, Zsolt Boldogkői^1,*^**

^1^Department of Medical Biology, Faculty of Medicine, University of Szeged, Szeged, 6720, Hungary

^2^Veterinary Diagnostic Directorate of the National Food Chain Safety Office, Budapest,1143, Hungary

^3^Department of Genetics, School of Medicine, Stanford University, Stanford, CA 94305, USA

*[boldogkoi.zsolt@med.u-szeged.hu](mailto:boldogkoi.zsolt@med.u-szeged.hu)

**Supplementary Note 1. Terminology of the listed transcripts**.

X2R= a given CDS

X2R – the most abundant transcript, probably the main transcript of a given CDS

X2.5R – genes in novel genomic position

X2R.5 - new embedded, potential protein coding transcript

X2R-AT – transcript isoform with alternative 3’ end, resulting a longer 3’ UTR (may contains dORF)

X2R-l – long transcript isoform with alternative TSS, resulting a longer 5’ UTR than the main transcript (these may include uORF)

X2R-s – short transcript isoform with alternative TSS, resulting a shorter 5’ UTR than the main transcript

nc-X2R – non-coding transcript isoform terminating before the stop codon of a known ORF or transcript without ORF

tr-X2R – truncated transcript isoform within a CDS containing no start codon

c-X2R-X1L - complex transcript isoform, spanning oppositely oriented ORFs

as-X2R - antisense transcript isoform, oriented oppositely to the given ORF

**Supplementary Note 2. Information for downloading and using the Geneious file.**

The whole transcriptome profile of the VACV available at F**igShare**: 10.6084/m9.figshare.10191230

The dataset can be view by using Geneious software which is available at <https://www.geneious.com/free-trial/>

Features found in literature were transferred from the VACV reference genome (NC_006998) to the LT966077 with the Geneious 10.2.6 Annotation Transfer tool.

***The following abbreviations and terms are used in the file:***

Anomalous TSS: RNA 5′ ends were mapped within ORFs and antisense to ORFs [15,19]

Cat box: CCAAT-box, upstream regulatory sequence of TSS [42]

CDS: coding sequence;

CDS-corr: correction of annotated ORF; based on the ribosome profiling data [12]

CDS-d: downstream frameshifting ORF; based on the ribosome profiling data [12]

CDS-n: previous unannotated non-coding region ORF; based on the ribosome profiling data [12]

CDS-u: upstream ORF; based on the ribosome profiling data [12]

CDS-tr: downstream truncated ORF or upstream non-frame shifting ORF; based on the ribosome profiling data [12]

Early Promoter motif: sequence motif (AAAANTGAAAANNA) of early gene’s promoters [14]

Early PAS motif: sequence motif (TTTTTNT) for polyadenylation site of early genes [15]

mRNA: LoRTIA annotated transcript

Late Promoter motif: sequence motif (TNNNNNNNNNTAAATG) of late gene’s promoters [19]

LoRTIA: Long-read RNA-Seq Transcript Isoform Annotator toolkit [36]

PAS: polyadenylation site

TIS: translational initiation site

TSS: transcriptional start site

VACV: Vaccinia Virus

**Supplementary Note 3. List of transcripts with unknown or non-validated TSSs.**

**ORFs without annotated TSS**: VACVWR_00030, VACVWR_00040, VACVWR_00050, VACVWR_00130, F10L, I5L, I8R, G1L, G6R, G7L, L3L, H2R, H4L, D2L, D3R, D6R, D10R, A7L, A11L, VACVWR_01470, A30L, A32L, A38L, VACVWR_01640, VACVWR_01870, B4R, VACVWR_01940, VACVWR_02050, VACVWR_02140, VACVWR_02150, VACVWR_02160

**Only Moss-lab**: VACVWR_00340, F3L, J5L, A24R

**Only our data**: VACVWR_00120, C3L, C8L, VACVWR_00430, F9L, F13L, F17R, E7R, E8R, E10R (??c)E11L, O2L, I1L, I2L, I6L, I7L, G2R (c), G8R, G9R, L1R, L4R, L5R, J1R, H1L, H3L, H6R, H7R, D11L, D13L, A1L, A2L, A2.5L, A3L, A6L, A9L, A10L, A12L, A13L, A14L, A14.5L, A15L, A16L (??A17L.5), A18R, A19L, A20R (?? c), A22R, VACVWR_01440, VACVWR_01450, VACVWR_01460, A26L, A27L, A28L, A31R, A34R, A39R, A43R, VACVWR_01840, B7R, VACVWR_01990, B18R, VACVWR_02040, VACVWR_0207, C13L, B25R, B28R
