# Supplementary material for: Long-read assays shed new light on the transcriptome complexity of a viral pathogen
Source: Sci Rep. 2020 Aug 14;10:13822. doi: 10.1038/s41598-020-70794-5 (PMC7427789; doi:10.1038/s41598-020-70794-5)
Supplement: Supplementary file 3 — Supplementary information 3 [file 41598_2020_70794_MOESM3_ESM.docx]

**Long-read Assays Shed New Light on the Transcriptome Complexity of a Viral Pathogen**

**Dóra Tombácz^1^, István Prazsák^1^, Zsolt Csabai^1^, Norbert Moldován^1^, Béla Dénes^2^, Michael Snyder^3^, Zsolt Boldogkői^1,*^**

^1^Department of Medical Biology, Faculty of Medicine, University of Szeged, Szeged, 6720, Hungary

^2^Veterinary Diagnostic Directorate of the National Food Chain Safety Office, Budapest,1143, Hungary

^3^Department of Genetics, School of Medicine, Stanford University, Stanford, CA 94305, USA

*[boldogkoi.zsolt@med.u-szeged.hu](mailto:boldogkoi.zsolt@med.u-szeged.hu)


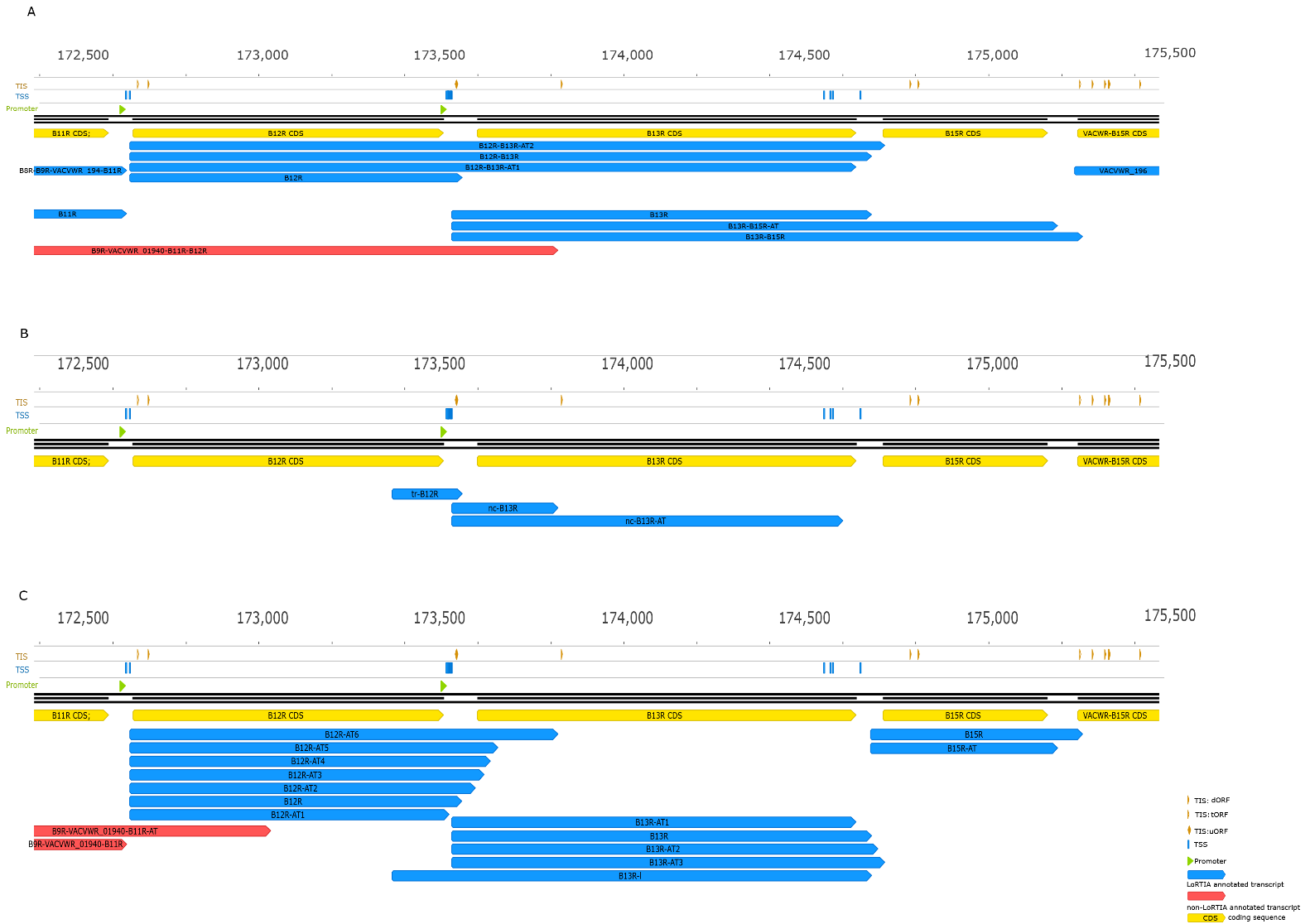


c

b

a

**Supplementary Figure S1. The B12R-B13R-B15R region of the VACV genome as a typical example of a regular transcript region in the VACV transcriptome.** (a) illustration of polycistronic transcripts within the B12R-B15R region; (b) examples of non-coding transcripts; (c) representation of monocistronic transcripts and 5’- and 3’-isoforms.


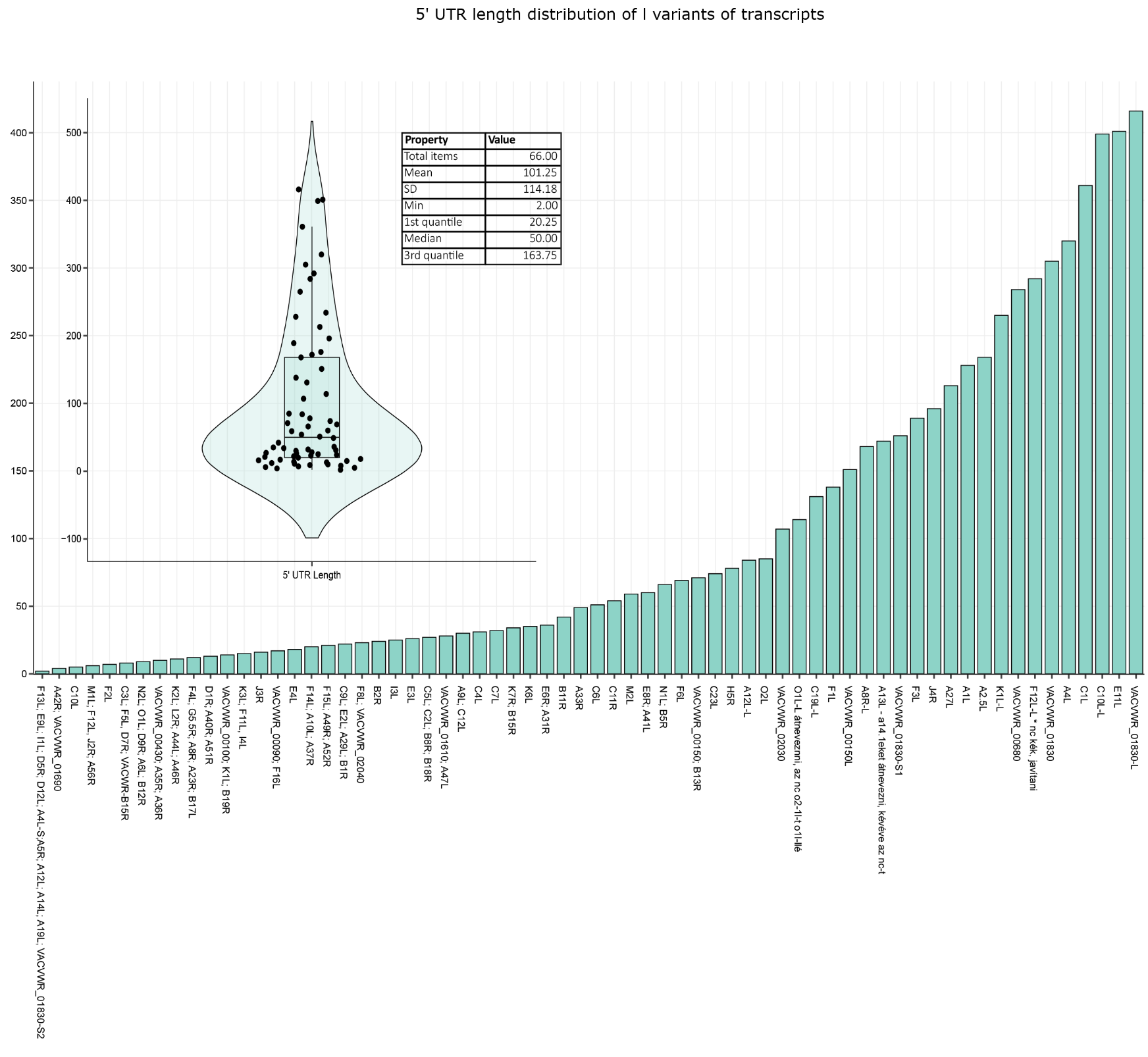


**Supplementary Figure S2.** 5'-untranslated region (UTR) length distributions of transcript variants. The column diagram and the violin plot show 5’-UTR lengths of VACV transcripts ranging from 2 to 400 nts.


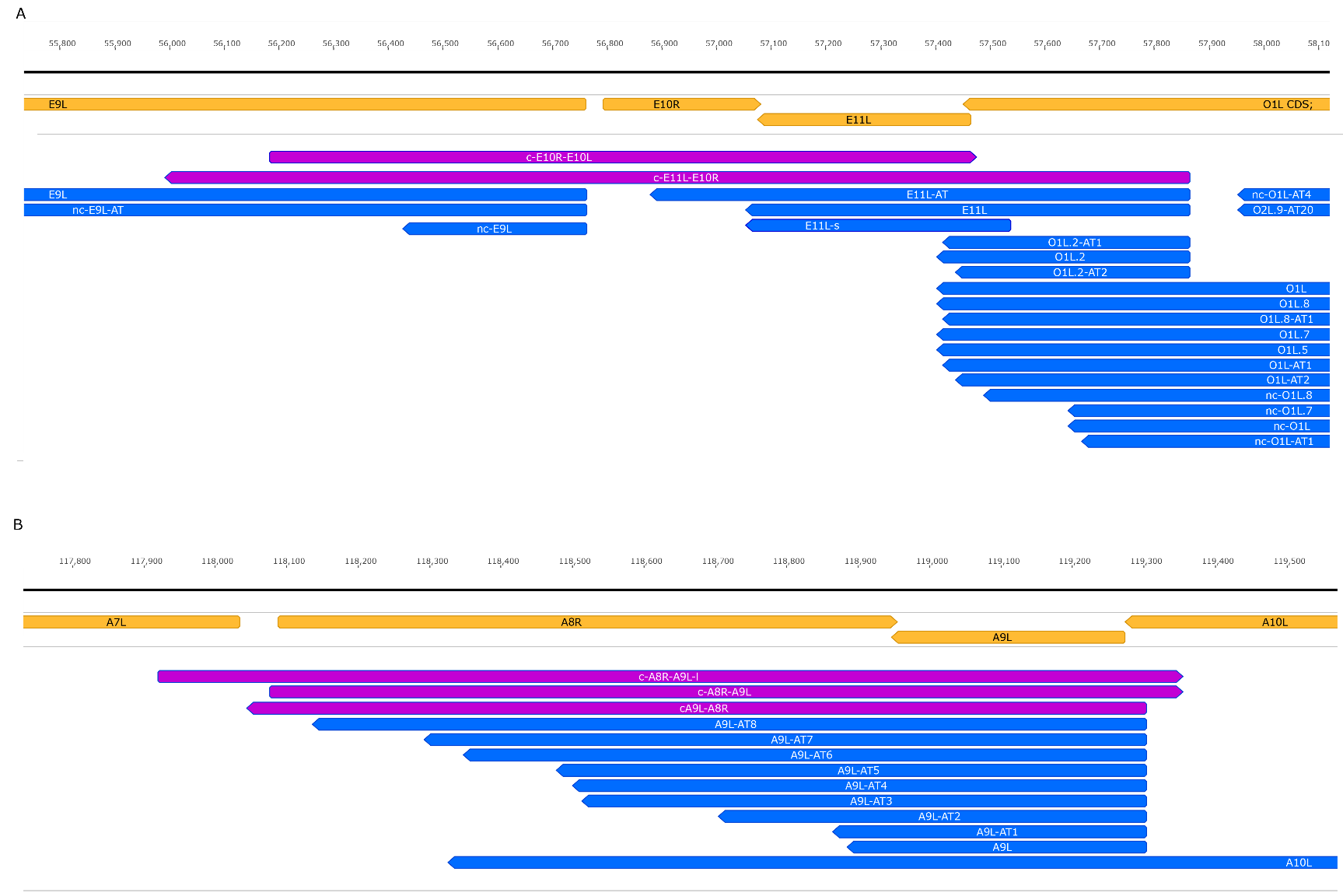


b

a

**Supplementary Figure S3.** Fully overlapping complex transcripts. (a) A8R-A9L genomic loci. (b) E10R-E11L region. Color legend: yellow: annotated ORFs; blue: transcripts, determined by this study; magenta/purple: cxRNAs


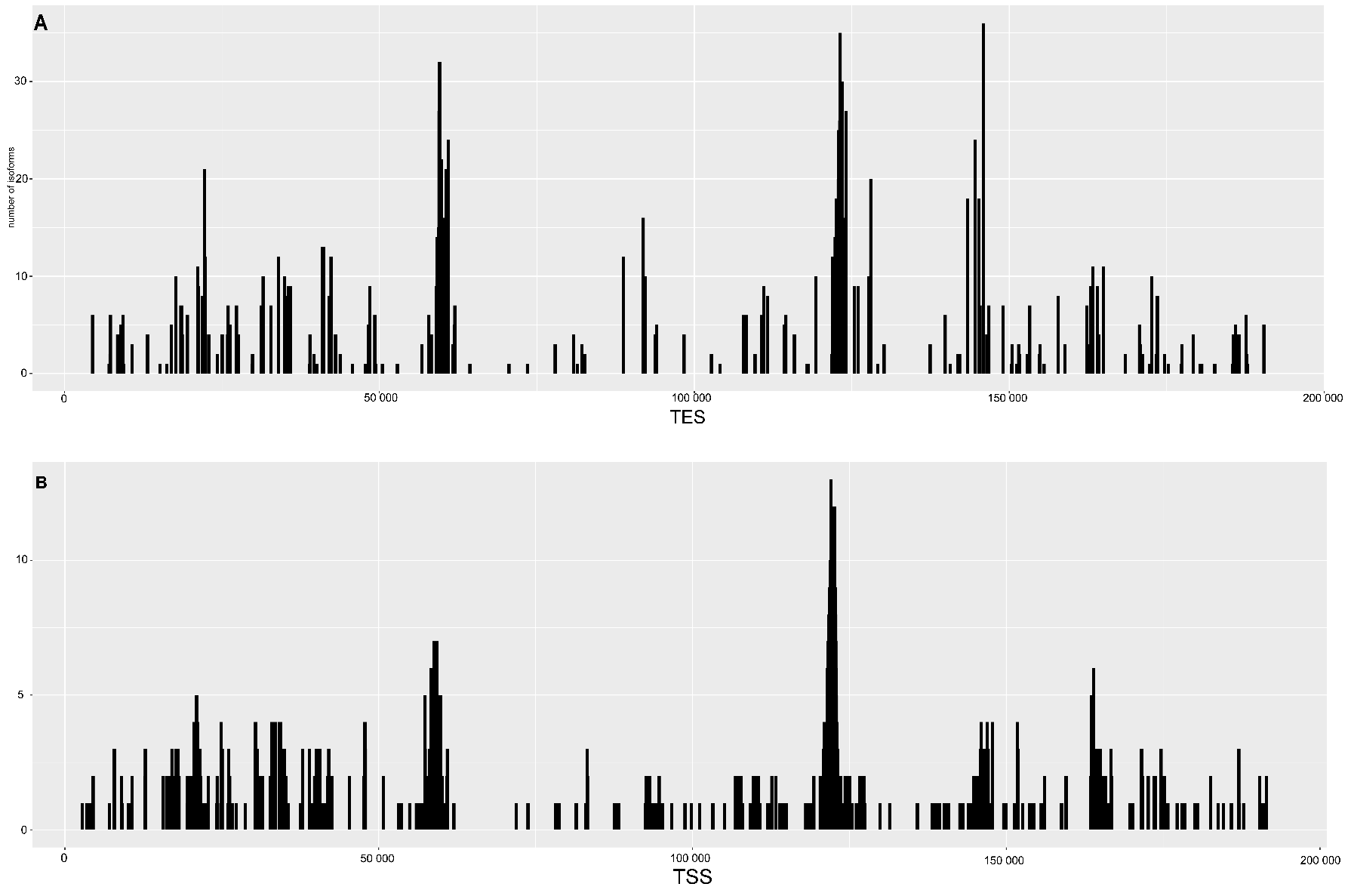


b

a

**Supplementary Figure S4.** Shared transcription start and end site positions across the VACV genome. (a) TSS sites shared by ≥ 1 transcript isoforms; the X axis represents genome positions and the Y axis shows the number of transcript isoforms. (b) TES positions shared by ≥ 1 RNA isoforms; the X axis represents the genome positions and the Y axis shows the number of transcript isoforms.


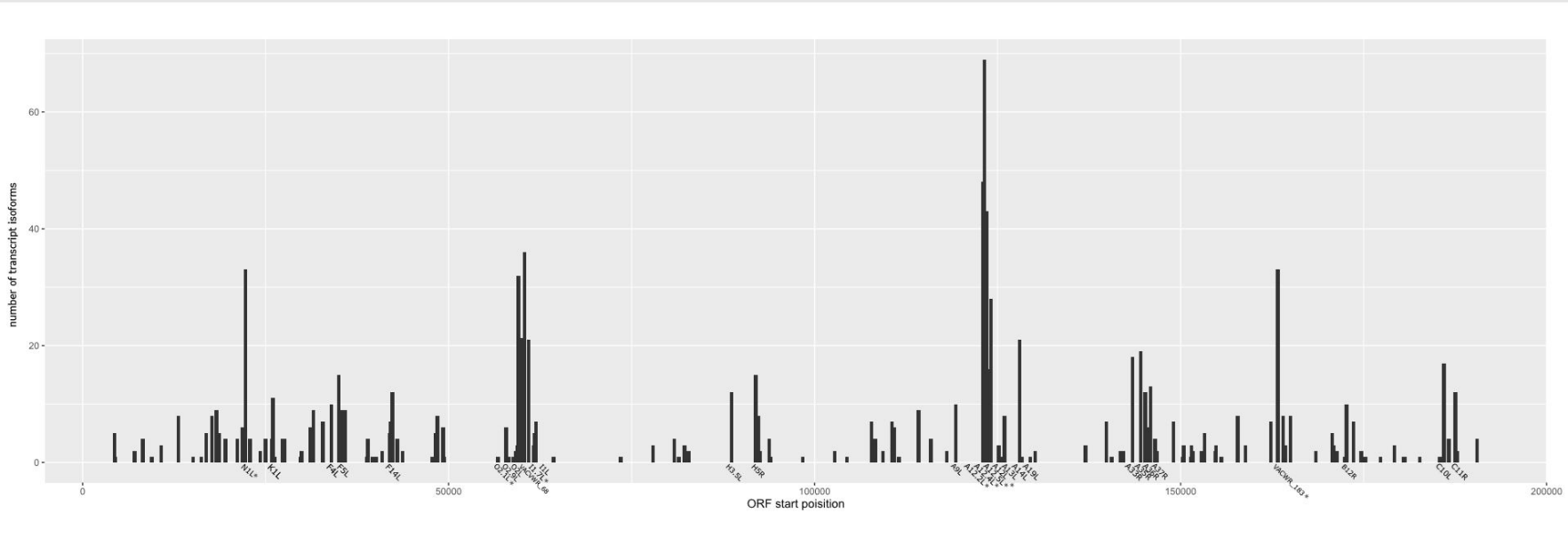


**Supplementary Figure S5.**Transcript isoforms. The X-axis represents genomic coordinates of the first ATGs of the ORFs. The number of LoRTIA-annotated transcript isoforms of given genes are shown on Y-axes.


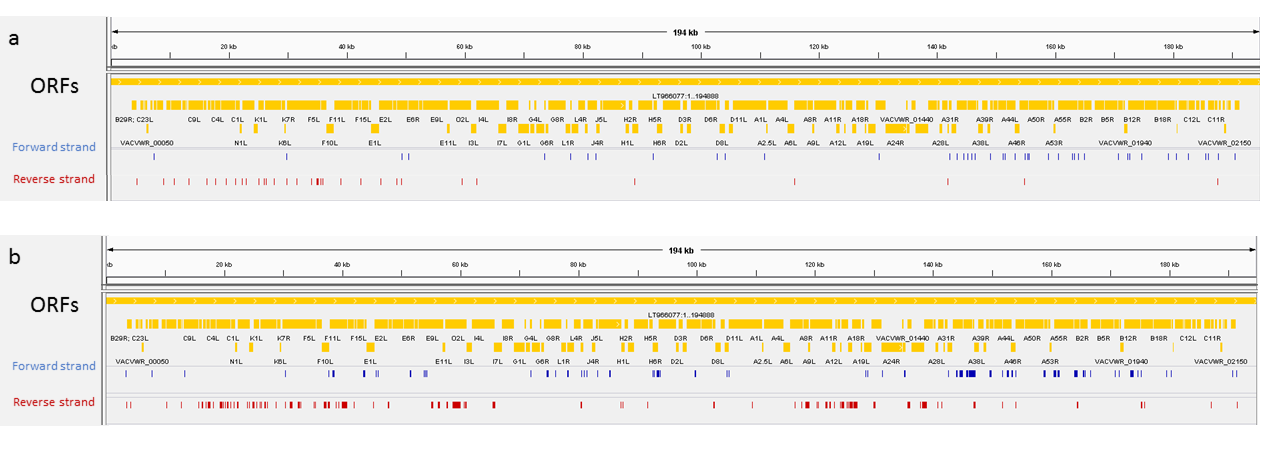


**Supplementary Figure S6.** Genome-wide distribution of VACV transcription start and end sites**.**


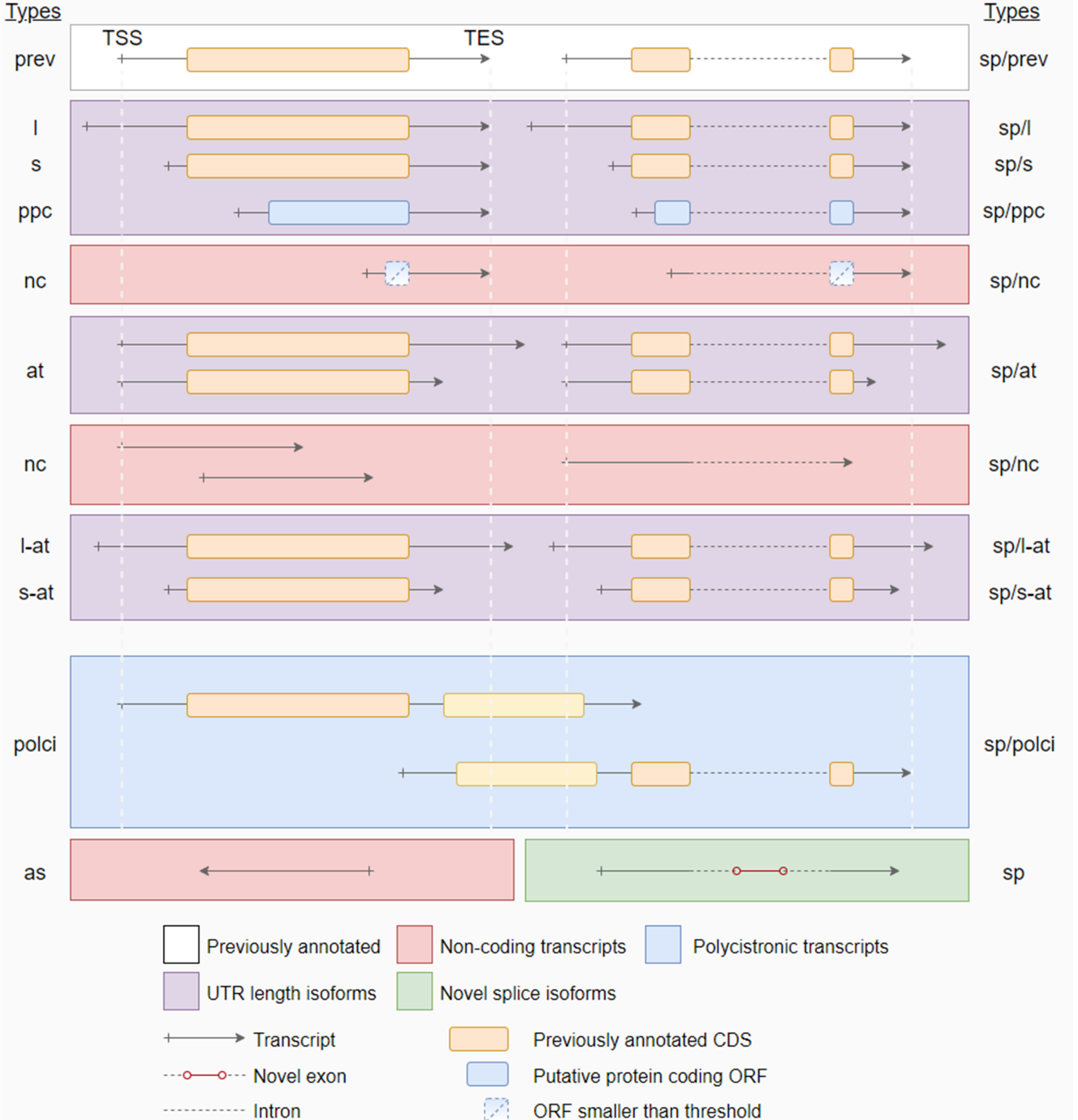


**Supplementary Figure S7.** The transcript isoform categories used in this study and their abbreviations are shown.
